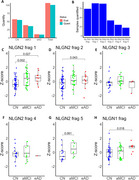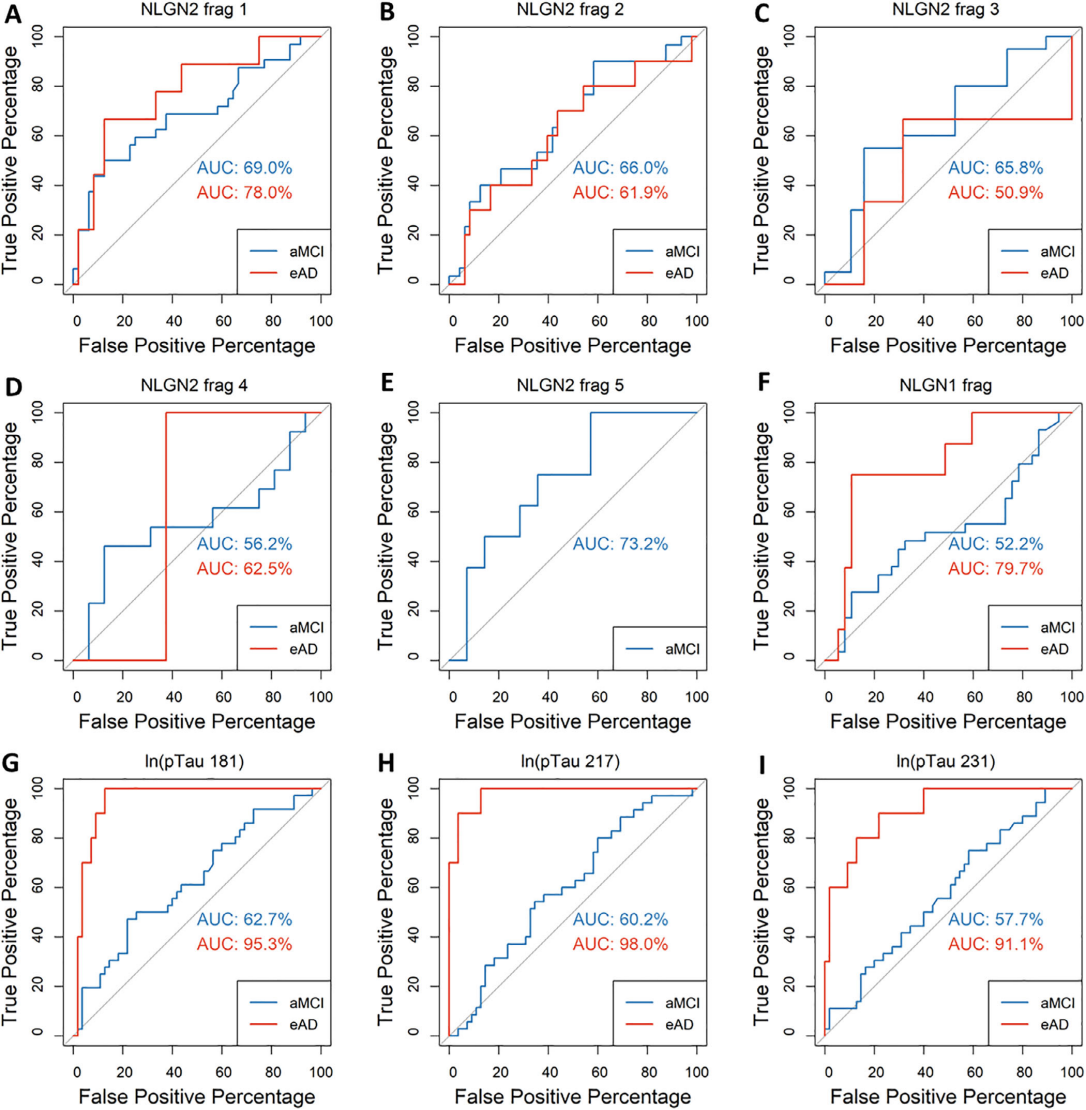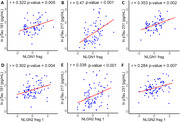# Neuroligin fragments as blood‐based biomarkers for early detection of Alzheimer's disease

**DOI:** 10.1002/alz70856_097403

**Published:** 2025-12-24

**Authors:** Milton Guilherme Forestieri Fernandes, Maxime Pinard, Esen Sokullu, Jean‐François Gagnon, Frederic Calon, Benoit Coulombe, Jonathan Brouillette

**Affiliations:** ^1^ Université de Montréal, Montreal, QC, Canada; ^2^ Institut de Recherches Cliniques de Montréal, Montreal, QC, Canada; ^3^ Centre for Advanced Research in Sleep Medicine, Hôpital du Sacré‐Cœur de Montréal, Montreal, QC, Canada; ^4^ Université du Québec à Montréal, Montreal, QC, Canada; ^5^ Laval University, Quebec, QC, Canada; ^6^ Centre de recherche du CIUSSS‐NIM, Montréal, QC, Canada; ^7^ Université de Montréal, Montréal, QC, Canada

## Abstract

**Background:**

Alzheimer's disease (AD) progresses slowly, with advanced neurodegeneration already present by the time clinical symptoms appear. To enhance the effectiveness of potential treatments, biomarkers that can detect the disease in its early stage are needed. Neuroligin (NLGN) is present in synapses, which are affected early in AD. Fragments of NLGN can be detected in the blood during the initial stages of the disease, suggesting their potential as biomarkers for early detection. In this study, we evaluated the potential of NLGN fragments as blood‐based biomarkers for the early stages of Alzheimer's disease.

**Method:**

Blood samples were obtained from the CIMA‐Q cohort, which includes individuals at preclinical and prodromal stages of AD, those in the early stages of the disease, and cognitively healthy individuals. To quantify the levels of NLGN fragments in blood, we employed multiplexing combined with high‐resolution tandem mass spectrometry. Statistical analyses were then conducted to examine differences in NLGN fragment levels across diagnostic groups and to assess their correlation with established indicators of AD progression, including plasma phosphorylated Tau (pTau) levels, hippocampal volume, and Mini‐Mental State Examination (MMSE) score.

**Result:**

The levels of certain NLGN fragments were elevated in individuals with amnestic mild cognitive impairment (aMCI), a prodromal stage of AD, and in the early stages of AD. A significant positive correlation was observed between plasma phosphorylated Tau (pTau) levels and specific NLGN fragments. Additionally, in non‐healthy individuals (aMCI and early AD), NLGN fragment levels showed an inverse correlation with MMSE scores and hippocampal volume.

**Conclusion:**

Identifying changes in synaptic proteins in the blood of individuals with aMCI and early AD could be instrumental for earlier disease detection. Our findings reveal that blood levels of specific NLGN fragments are elevated in the prodromal and early stages of AD and correlate with key indicators of disease onset, particularly plasma levels of pTau. These results suggest that NLGN fragments could serve as a primary care screening test and be incorporated into a panel of early biomarkers of AD.